# A mitogenomic phylogeny of pierid butterflies and complete mitochondrial genome of the yellow tip *Anthocharis scolymus* (Lepidoptera: Pieridae)

**DOI:** 10.1080/23802359.2020.1781578

**Published:** 2020-06-24

**Authors:** Yan Zhou, Can Zhang, Shaoquan Wang, Yanlin Liu, Ning Wang, Bin Liang

**Affiliations:** a Co-Innovation Center for Sustainable Forestry in Southern China/College of Biology and the Environment, Nanjing Forestry University, Nanjing, China; b Department of Eco-Engineering, Guangdong Eco-Engineering Polytechnic, Guangzhou, China; c Chinese Felid Conservation Alliance (CFCA), Beijing, China; d Department of Ornithology, American Museum of Natural History, New York, NY, USA; e Hainan Academy of Forestry, Haikou, China

**Keywords:** Mitochondrial genome, Mitogenomic phylogeny, Pieridae, *Anthocharis scolymus*

## Abstract

The yellow tip butterfly *Anthocharis scolymus* (Lepidoptera: Pieridae) has a circular mitochondrial genome of 15,230 bp in size. It consists 13 protein-coding genes, 22 tRNAs, two ribosomal RNA genes, and an AT-rich control region. Using whole mitogenome alignments, we reconstructed the phylogenetic relationships of 28 pierid butterflies. The maximum-likelihood (ML) tree topology was consistent with previous studies.

The Pieridae, one of the largest butterfly families, contains approximately 83 genera and over 1100 species worldwide (Braby 2005). This family is currently classified into four monophyletic subfamilies (Dismorphiinae, Pseudopontiinae, Coliadinae, and Pierinae) (Braby and Trueman 2006; Wahlberg et al. 2014). The genus *Anthocharis,* which was included within the subfamily Pierinae, consists about 15 species, distributed in North Africa, Eurasia, and North America (Zhou 2000; Okumura et al. 2016). Recently, the accumulation of whole mitochondrial genomes has facilitated phylogenetic studies in insects (Cameron 2014; Liang et al. 2018; Zhou et al. 2020). Here, we presented the first complete mitogenome of the yellow tip (*Anthocharis scolymus*), which inhabits mainland China, Far East Russia, the Korean Peninsula, and the Japanese Archipelago in East Asia (Kinoshita 1998). Moreover, we present the largest mitogenomic phylogeny of pierid butterflies so far, which would contribute to studies of mitogenomic evolution and phylogeny in butterflies.

An individual male *A. scolymus* was collected from the campus of Nanjing Forestry University (E118.82, N32.08), Nanjing, Jiangsu Province, China on 16 April 2020 (voucher number: BL_YZ_HJJFD_003, kept in College of Biology and the Environment, Nanjing Forestry University, Nanjing, China). Total genomic DNA was extracted and we followed the major protocol in our previous study (Chen et al. 2020) for genomic library construction and sequencing. The generated reads were filtered, trimmed, and mapped to reference mitogenome of *A. bambusarum* (GenBank: NC_025274) and then assembled using Novoplasty3.8.3 (Dierckxsens et al. 2017). The average sequencing coverage is about 800×. We performed automatic annotation using the MITOS web server (Bernt et al. 2013) and manually verified the start and stop codons of genes by aligning and comparing with closely related species.

To explore the phylogenetic position of *A. scolymus*, we collected mitogenomes of 27 additional pierid butterfly species and five outgroups from Genbank. After excluding the control region from the whole mitogenomic alignments, a maximum likelihood (ML) tree was built in RAxML v8.2.10 (Stamatakis 2014) with the GTRGAMMA model and 200 ultrafast bootstraps (-f a). The ML tree highly supported (100%) a sister relationship between *A. scolymus* and *A. bambusarum* (Figure 1), and a monophyletic clade of all pierid butterflies. The overall topology is also consistent with previous reports (Espeland et al. 2018; Liu et al. 2020).

**Figure 1.**
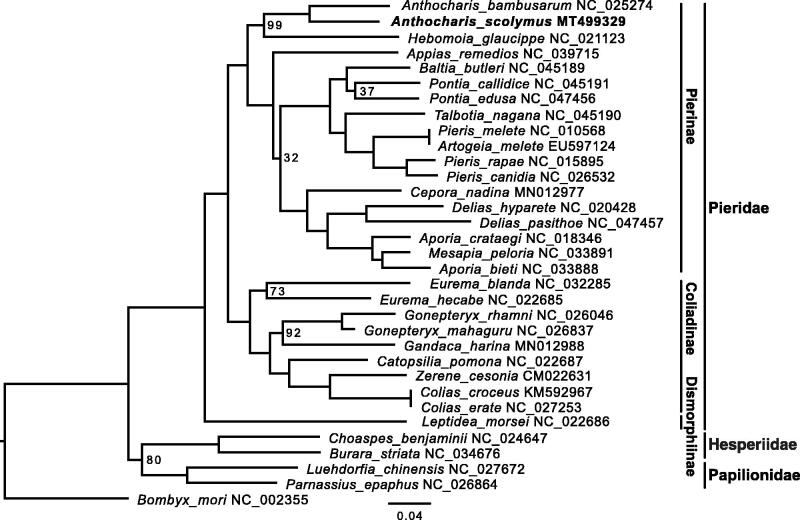
Inferred phylogenetic relationships among pierid butterflies based on whole mitogenome alignment excluding the extremely gappy control region. The domestic silkworm *Bombyx mori* was used as the outgroup. Bootstrap value at nodes is 100% unless indicated on the tree. GenBank accession numbers of all species used in this study are shown by the species name.

The complete mitochondrial DNA of *A. scolymus* is a circular molecule of 15,230bp in length with 19.5% GC content. It encodes 37 genes including 13 protein-coding genes, the standard 22 tRNAs, two ribosomal RNA genes, and a putative control region (GenBank accession number: MT499329). The characteristics of the gene order and intergenic spacers in the *A. scolymus* mitogenome are the same as that of other pierid butterflies (Cao et al. 2016).

## Data Availability

The data that support the findings of this study are openly available in GenBank of NCBI at https://www.ncbi.nlm.nih.gov, reference number MT499329.
